# Middlesex Hospital

**Published:** 1829-09

**Authors:** 


### HOSPITAL REPORTS,

(*Principally condensed from various Periodical Publications.*)

### MIDDLESEX HOSPITAL.

*Case of Traumatic Tetanus, successfully treated.*

We have recently witnessed, with considerable interest, the recovery of a patient from traumatic tetanus, in which the principal remedy employed was calomel. The amendment took place on the mouth becoming affected. The following are the particulars of the case.

John Kelly, ætat. fourteen, was admitted into the Middlesex Hospital, under the care of Mr. Mayo, on the 1st of July, having fallen from a scaffolding; by which accident the radius of the right arm was broken near the wrist, and the integuments of the right knee were lacerated and torn back. Nothing unfavorable occurred, till more than a fortnight had elapsed; at which time the wound of the integuments was almost healed; but, on the 17th, towards evening, the lad complained of stiffness of the jaws and of the back of the neck, of which he now said he had felt something the preceding day. The house surgeon directed the application of a blister to the back of the neck, and a purgative enema.

On the 18th, at twelve o'clock, Mr. Mayo saw the patient. At that time the jaw did not admit of being depressed above one third of an inch; the back of the neck, the back, and abdomen were rigid; and the permanent spasm was occasionally heightened by a brief and more violent action of the muscles; the countenance was anxious and alarmed, and bathed in perspiration; the tongue furred, but moist; pulse 150; bowels confined. Sixteen leeches were applied to the back of the neck, and six grains of calomel administered; and shortly afterwards two drops of oil of croton.

Four o'clock.—The bowels have acted twice and copiously. It was now Mr. Mayo's intention to try the carbonate of iron: accordingly, a drachm of this medicine was given to the lad; but, as he swallowed with great difficulty, even this quantity of the remedy was some time in being got down; and, although half a drachm of laudanum was given with it, the dose was speedily returned by vomiting. Under these circumstances, the medicine was directed to be changed. At seven o'clock the patient took ten grains of calomel, and the same dose at eleven o'clock, with a drachm of laudanum. At this time the tongue had become dry, but the pulse had fallen in frequency, and the lad appeared to swallow more easily. Poultices, containing Goulard's lotion and laudanum, were applied to the unclosed, but not unhealthy, wound on the knee, and to the blistered surface at the back of the neck.

19th.—The lad slept occasionally during the night. There was no essential alteration in his appearance. At noon he took five grains of calomel, and two of tartarized antimony, which were repeated every two hours; but in the evening he became sick, and vomited, and the calomel alone was continued.

20th.—His appearance this morning was changed for the worse: the jaws were closer, and the muscles more rigid. He was taken into the bath-room, and three pails of cold water were thrown over him. His pulse sunk temporarily to ninety, and was irregular. He experienced some slight but temporary relief: he was, he said, "fresher and better," and had no objection to the repetition of the cold affusion. At three it was repeated, but without benefit. At night he took a grain of opium and a grain of acetate of lead; which dose was repeated once the following morning.

21st.—He has again had some sleep during the night; but the general spasm of the muscles of the jaws, neck, and trunk remains. The extensor muscles have the advantage, and keep his body in a position approaching to opisthotonos.—℞. Hydr. Submur. gr. iij.; Antim. Tart. gr. i.; Pulv. Opii gr. ss. every three hours.

22d.—He is distinctly better; the mouth admits of being opened wider. At the same time he is largely purged, and the breath has the mercurial fetor; the gums are tender, the cheeks sore.

From this time the lad recovered rapidly; the mercury being discontinued, and his strength gradually restored by nourishment cautiously given.

There were two circumstances worthy of observation during his recovery: the spasm of the muscles did not disappear at once, but was each day sensibly less than on the preceding. On the 3d of August, it was the boy's impression that he had completely got rid of the stiffness about his neck and jaws. The other circumstance was the following: One evening, about the 27th, while there was great rigidity of the body yet remaining, Mr. Mayo, on visiting his patient, found him asleep, and remarked that he lay perfectly relaxed; the abdominal muscles were soft and yielding, and had not the least tension. The boy was wakened, and at the instant the full tension of the muscles returned. Not being further disturbed, he fell asleep in a few minutes, when the muscles again became relaxed; and again, on his being wakened, resumed the state of spasm.

---

*Laceration of the Perinæum and Sphincter Ani. Involuntary Discharge of Fæces. Operation performed with success.*

Charlotte Kendall, æt. twenty-five, was admitted into the Middlesex Hospital, under the care of Mr. Mayo, about the middle of May. She had been confined, for the first time, on the 19th of the preceding October. The labour was not severe; but two days afterwards she observed that the fæces passed away involuntarily. This distressing circumstance continued; and, at her admission, she mentioned that the period of twenty-four hours, at which it habitually occurred, was very regular: from five in the morning till eleven in the forenoon, the bowel used at intervals to discharge its contents, and not during the rest of the day or the night.

On examining the parts, the perinæum appeared to have been extensively lacerated, and the sphincter entirely torn through into the vagina.

The operation of paring the edges of the laceration, and sewing them together with four stitches, was then performed. On the third day some opening medicine was administered, when the ligatures gave way, and the fissure became as before.

On the 17th of June, Mr. Mayo repeated the operation. For the nine subsequent days the patient was kept perfectly quiet, with very little nourishment, and without medicine. For this period there was no uneasiness, and no action of the bowels. Motions were then obtained by means of castor oil and an enema; when it seemed at first to the patient that nothing had been gained by the operation. In a day or two, however, she found that she certainly had acquired *some* control over the action of the bowels. Then an attack of diarrhœa ensued, and every thing again came away uncontrollably; but, on her recovery from this attack, it became evident that a real and important improvement in the state of the parts had taken place. It now appeared to her that the sphincter had been restored; and, on examining the parts, it was found that almost the whole of the fissure had united. After the experience of four or five weeks, she ascertained that, as long as the motions were not relaxed, she had perfect and entire control over the bowel: when the motions are loose, on the contrary, they still come away involuntarily.

Upon weighing these circumstances, Mr. Mayo, in our opinion very judiciously, recommended his patient to be contented with the advantage that had been gained, and not to risk its loss by a repetition of the operation.

